# Supplementary material for: Sex differences in task engagement and lapse rate during reward learning
Source: Oxf Open Neurosci. 2026 Jun 13;5:kvag004. doi: 10.1093/oons/kvag004 (PMC13280948; doi:10.1093/oons/kvag004)
Supplement: Supplementary_Materials_kvag004 [file supplementary_materials_kvag004.zip › Review history for OXFNSC-2025-003.R2.odt]

Review history for OXFNSC-2025-003.R2

Round 1 reviews
Reviewer 1
Recommendation – Reject
Comments to the Author
In this manuscript the authors trained male and female rats to perform a stimulus-based (the identity of the stimulus cued reward) or action-based reversal learning task (the location of the stimulus cued reward). Rats were trained in a deterministic version of the task, followed by two reversals and finally two more reversals where reward delivery was probabilistic. They then compare a variety of performance metrics between males and females and use a reinforcement learning model to compare trial-by-trial learning and fit a sigmoid to the choices after reversal to characterize the behavioral reversal. They find modest differences in choice behavior and larger differences in the tendency of males and females to omit trials and in the latency to initiate trials when they don’t omit.
The study of sex differences in behavior is important and generally understudied, although comparisons in this type of task have been conduced (e.g., Chen et al 2021). The differences in trial omissions and latencies are convincing and consistent with previous reports. Regarding the choice analyses, however, there are some problems with the interpretation of the behavioral modeling and statistics that make their meaningfulness unclear.

1. In general, the design of the mixed effects regressions should be improved. In some cases, it is not clear why particular sets of trials (e.g., incorrect and correct or sessions around each reversal) were fitted separately. Why not just include regressors for those conditions and include interactions with sex? This provides is a clearer comparison of conditions and helps avoid the statistical pitfall of condition A and B are different because there’s an effect in A but not B. Some specific examples are below:
A. In figure 2, there should be an interaction between sex and trial/session to evaluate differences in learning/reversal rates. Evaluating a main effect of sex will only account for overall differences in performance but not a change in the learning rate. Indeed, this is clear from 2E where there is a small shift in performance between males and females (males performing better) which was captured by the reported main effect of sex. But this does not mean that females learned the reversal more slowly than males as stated on line 343-345. Similarly, the authors report no differences in the action-based version because there was no main effect of sex (Figure 2B), but it does look like there could be a small difference in the slopes of the performance curves that may be captured with an interaction.
B. In figure 4, it is not statistically rigorous to conclude that the sex difference in omissions occurs in late but not early learning because there is an effect in one condition but not the other (see https://elifesciences.org/articles/48175). The effect would need to be explicitly compared in the early and late stage. One way to do this would be to include the interaction between sex and session in this analysis. A significant interaction would indicate that the sex difference is dependent on stage. Basically, instead of fitting 3 (?) models (all sessions, session 1 and session 2 or 5) the authors could fit a single model that included regressors for sex (to capture the average difference), session and sex*session (to determine if the sex difference depends on session). Post hoc tests can then be used to determine which stage differed between males and females.
2. It does not appear that males or females learned the stimulus-based contingencies in the probabilistic condition (and barely learned the initial task or the deterministic reversals). It’s not reported, but I would be surprised if performance is significantly different from chance in 2f, which makes comparison of learning less meaningful (i.e., if nobody learned then no one learned faster or slower). It’s stated in the methods that rats were given 10 sessions to reach criterion when learning the stimulus-based version of the task. The authors should show how many rats actually reached criterion and as mentioned above report whether performance differenced significantly from chance. I’m not sure that we can draw conclusions from these data at all unless there’s something interesting to deduce from the fact that female rats are more likely to omit/slower to initiate trials even in a condition where they have not learned the task.
3. I’m confused about the interpretation of the lapse rates in figure 5. It is stated in the text that e1 is larger than e2 which indicates that the rats make more errors prior to the transition. But doesn’t a higher lapse rate mean that they are selecting the worse offer less often (e.g., making fewer mistakes) than if they had a smaller lapse rate? Maybe the definition of errors in this case needs to be expanded a bit. I would think that every “worse” choice after the reversal is an error and the lapse rate prior to the behavioral transition captures the extent to which the animals are not making that choice.
4. I also don’t think that including the omit trials makes sense or if they are included, it doesn’t seem justified to interpret them as a choice halfway between right and left in this analysis. These are likely lapses of engagement and are therefore hard to interpret as a choice between the two options. It’s also a big assumption that the rats would have been equally likely to choose left or right if they had made a choice on those trials. There could be structure in the omissions (e.g., are they more likely after unrewarded trials? There is clearly structure in the choice across a session too in that they are more likely to make a the “better” choice late in the session). Including these trials in this way could also be a problem for interpretability given that females omit more trials than males. Are the differences between males and females really about how long females perseverate on the old option (which would lead to a larger post-transition lapse rate than males) or how often they omit? It’s also not clear how they were included anyway – the description of the model describes binomial data. Comparing the performance after the transition between males and females helps with the point the authors are trying to make (although the test is convoluted – why not just compare performance after the behavioral transition rather than comparing the differences in performance before and after the transition?)
5. The authors could do a better job justifying the behavioral modeling and why it is informative to compare them. Are they trying to argue that rather than incremental trial by trial updating (as captured with the RL model) the behavior is better explained as a state transition (as captured with the sigmoid fits)? I’m not sure that these hypotheses can be clearly distinguished in a deterministic task because once the animal starts choosing the high reward option they will always be rewarded so the value for that choice will never be reduced if it is chosen. So effectively, this would look like a transition. A test of this would be to use the RL model to simulate choices during reversals (so the mechanism of updating is known) and then compare performance of the two models on the simulated data. My guess is that they’d be pretty similar.
6. Line 459: poorer choice accuracy in females after the transition wasn’t tested


Minor:
1. Is there a typo on line 195? Elsewhere it says the low probability option was rewarded 10% of the time rather than 0%.
2. When describing the sigmoid fits at reversals, I think it would be clearer to refer to “behavioral transition” instead of just “transition” since this could be confused with reversal which has already happened.
3. Fitting a sigmoid to choice around transitions isn’t a Hidden Markov Model. The paper that is cited used a Hidden Markov Model to infer transitions between states defined by the fit of the logistic regression around reversals (so the sigmoid fits were different in each state and the transitions between those states were described by the HMM rather than the transition from low to high choices at reversals).
4. Was the sex*reversal interaction on omissions, trial initiation latencies and earned rewards described on line 369-371 (also, this is the sort of model design I described above – include the interaction and then use the specific post hoc comparisons to describe how the sex difference changes across conditions) assessed for the action-based task?
5. On line 399 the authors say that they do not include the stimulus-based sessions in the RL model fitting because the fit is poor but then there’s a section about it starting at line 436.

Reviewer 2
Recommendation – Major Revision
Comments to the Author:
The authors present new results comparing sex differences in decision making strategies. Here, the authors use a previously published paradigm to study sex differences during reversal learning in both an action and stimulus domain. Using mixed-effects models, reinforcement learning models, and state transition models, they find that female rats are more likely to omit trials, take longer to forget the value of unchosen options, and have a higher lapse rate after choice transitions, while male rats were more likely to utilize a Win-Stay strategy in action-based reversal learning.

Overall, I feel like the results of this study are a useful contribution to an under-explored area of animal decision making; however, I would suggest additional clarification and explanations to make the manuscript stronger.

High-level comments:
- The results section is sometimes lacking motivation for why specific measures are chosen. For example, why are initiation latencies, correct choice latencies, reward latencies, etc., being compared? What are these metrics meant to evaluate? Much of this could be bolstered by using a results format like: "First we wanted to test if X is different between male and female rats. To measure this, we used Y. The results show Z." This is already being done in some places, e.g. for "Action-based learning" on line 385, we see the motivation with the sentence: "To capture animals’ sensitivity to positive and negative feedback, we assessed their use of win-stay and lose-shift adaptive strategies in action-based reversal learning."
- There are some prior results mentioned in the introduction that seemingly conflict with some of the results seen in this study, e.g. female mice displaying more Win-Stay strategies in a two-arm bandit, and female mice being more sensitive to negative feedback. I don't think the disparities are problematic, but I believe it should be addressed and compared further in the results section.
- The conclusions feel fairly generic. It would be nice to have the conclusions be more tailored to what was discovered here, what questions it raises, how it fits into a broader picture, and how they would inspire follow up studies. It's unclear what chronic stress has to do with this paradigm, also there's the mention of previous studies' results showing sensitivity to negative feedback but no connection to what was seen in the present study.
- I leave this up to the authors' discretion, but I believe it would be useful to try applying a mixture-of-agents approach to modeling RL (e.g. Miller et al., 2017, Nat. Neuro.; Akam et al., 2021, Neuron) and comparing it with other potential strategies, such as a Win-Stay Lose-Shift or a perseveration strategy. Additionally, this might help provide a better model fit to the stimulus-based reversal learning task, where they don't seem to be using a RL strategy at all.

Results-level comments:
- While I gleaned that the infusion was added to be able to include the additional control animals from a previous study, the results section lacks any mention of controlling for the role of infusions. Furthermore, the methods section states on line 106: "we entered drug as a covariate in order to account for any potential behavioral effects following CNO administration". However, the mixed-effects model later on does not include a factor for drug, so it is unclear how it is being controlled for in the main findings. Finally, it took a couple reads to really realize that it was only the control animals being used from the previous study, and I assume the injection protocol here is following the control protocol from the previous study, so I assume that the injections are not meant to have an effect. It would be nice for the purpose of the injections to be more clearly laid out, as well as to have a supplemental figure demonstrating that the infusion was controlled for and ideally does not impact the results seen in the main text.
- There are a number of trial-based metrics that would be strengthened by instead using or including trial count normalized metrics. For example, female rats were slower to initiate trials and collected fewer rewards, but is the latter because they are completing fewer trials? Is the reward rate per completed trial significantly different from males?
- The learning curves for the stimulus-based task are somewhat concerning seeing as how they never surpass chance levels. Is this consistent with previous studies using this task? It would be useful to have a supplemental figure with the learning curve during training, since the criteria suggests that they need to reach at least 75% performance before a reversal. Although there is the additional statement in the methods that they were also advanced after 10 days -- did any of the rats every meet the 75% threshold?

Line-specific comments:
- Line 153: use either "minutes" or add a period for the abbreviation "min."
- Line 228: missing closing bracket
- Line 365: typo "continency"
- Line 399: Statement about stimulus-based reversal learning is redundant with following section
- Line 436: underscore after paragraph heading 

Decision letter

Thank you for submitting Manuscript ID OXFNSC-2025-003 entitled "Sex differences in task engagement and lapse rate during reward learning plateaus" to Oxford Open Neuroscience.  Your manuscript was evaluated by two reviewers and their comments are included at the bottom of this letter.

Both reviewers agree that this work contributes to an under-studied and important topic. They have made several suggestions for clarifications and raised questions about the statistical basis for some of the conclusions. Therefore I am inviting you to submit a revision that responds to these points. Where additional statistical treatment is recommended, I will leave it to you to decide between conducting additional tests or amending conclusions where needed.

Please note that this journal operates with transparent peer review. This means that if your submission is accepted for publication, the full peer review history of your article will publish online alongside your article. This includes reviewer comments, editor decision letters, and your author responses.

Please revise your manuscript within the next 60 days from today date, or contact us if you would like an extension to this.

To revise your manuscript, log into https://mc.manuscriptcentral.com/oxfnsc and enter your Author Centre, where you will find your manuscript title listed under "Manuscripts with Decisions."  Under "Actions," click on "Create a Revision."  Your manuscript number has been appended to denote a revision.

You may also click the below link to start the revision process (or continue the process if you have already started your revision) for your manuscript. If you use the below link you will not be required to login to ScholarOne Manuscripts.

*** PLEASE NOTE: This is a two-step process. After clicking on the link, you will be directed to a webpage to confirm. ***

https://mc.manuscriptcentral.com/oxfnsc?URL_MASK=29dfbfc0fcb1472da9f4ef807918d364

You will be unable to make your revisions on the originally submitted version of the manuscript.  Instead, revise your manuscript using a word processing program and save it on your computer.  Please also highlight the changes to your manuscript within the document by using the track changes mode in MS Word or by using bold or colored text.

Once the revised manuscript is prepared, you can upload it and submit it through your Author Centre.

When submitting your revised manuscript, you will be able to respond to the comments made by the reviewer(s) in the space provided.  You can use this space to document any changes you make to the original manuscript.  In order to expedite the processing of the revised manuscript, please be as specific as possible in your response to the reviewer(s).

IMPORTANT:  Your original files are available to you when you upload your revised manuscript.  Please delete any redundant files before completing the submission.

Because we are trying to facilitate timely publication of manuscripts submitted to the Oxford Open Neuroscience, your revised manuscript should be uploaded within the next 60 days from today date.  If it is not possible for you to submit your revision in a reasonable amount of time, we may have to consider your paper as a new submission.

Once again, thank you for submitting your manuscript to Oxford Open Neuroscience and I look forward to receiving your revision.


Authors’ response
We thank the Editor and both reviewers for their constructive and helpful feedback. We have addressed their comments below and marked all changes in blue in the revised manuscript.

Reviewer: 1

Comments to the Author
In this manuscript the authors trained male and female rats to perform a stimulus-based (the identity of the stimulus cued reward) or action-based reversal learning task (the location of the stimulus cued reward). Rats were trained in a deterministic version of the task, followed by two reversals and finally two more reversals where reward delivery was probabilistic. They then compare a variety of performance metrics between males and females and use a reinforcement learning model to compare trial-by-trial learning and fit a sigmoid to the choices after reversal to characterize the behavioral reversal. They find modest differences in choice behavior and larger differences in the tendency of males and females to omit trials and in the latency to initiate trials when they don’t omit.

The study of sex differences in behavior is important and generally understudied, although comparisons in this type of task have been conducted (e.g., Chen et al 2021). The differences in trial omissions and latencies are convincing and consistent with previous reports. Regarding the choice analyses, however, there are some problems with the interpretation of the behavioral modeling and statistics that make their meaningfulness unclear.

1. In general, the design of the mixed effects regressions should be improved. In some cases, it is not clear why particular sets of trials (e.g., incorrect and correct or sessions around each reversal) were fitted separately. Why not just include regressors for those conditions and include interactions with sex? This provides is a clearer comparison of conditions and helps avoid the statistical pitfall of condition A and B are different because there’s an effect in A but not B. Some specific examples are below:

We thank the reviewer for this suggestion: we agree it would be a better practice to include session as a moderator in all GLM formulas to assess learning. We have corrected the formulas. See responses to specific examples below.

A. In figure 2, there should be an interaction between sex and trial/session to evaluate differences in learning/reversal rates. Evaluating a main effect of sex will only account for overall differences in performance but not a change in the learning rate. Indeed, this is clear from 2E where there is a small shift in performance between males and females (males performing better) which was captured by the reported main effect of sex. But this does not mean that females learned the reversal more slowly than males as stated on line 343-345. Similarly, the authors report no differences in the action-based version because there was no main effect of sex (Figure 2B), but it does look like there could be a small difference in the slopes of the performance curves that may be captured with an interaction.

As suggested, we have now included session as a moderator instead of as a covariate in all GLM analyses, allowing session to interact with sex. With these new formulas as the basis of our analysis (detailed in Data Analyses) there was no sex differences in learning and no interaction of sex by session in discrimination or reversal learning, in either domain. Thus, we have removed any statement that females learn more slowly than males. We think this makes the finding of sex differences in performance measures even more interesting. We have also changed the plots of learning in Figure 2 to collapse across reversals so the pattern of learning could be better appreciated. We thought this visualization was particularly useful for stimulus-based learning to show the modest gains from session 1 to later session 5, with the emphasis on completely overlapping datapoints between males and females.

B. In figure 4, it is not statistically rigorous to conclude that the sex difference in omissions occurs in late but not early learning because there is an effect in one condition but not the other (see https://elifesciences.org/articles/48175). The effect would need to be explicitly compared in the early and late stage. One way to do this would be to include the interaction between sex and session in this analysis. A significant interaction would indicate that the sex difference is dependent on stage. Basically, instead of fitting
3 (?) models (all sessions, session 1 and session 2 or 5) the authors could fit a single model that included regressors for sex (to capture the average difference), session and sex*session (to determine if the sex difference depends on session). Post hoc tests can then be used to determine which stage differed between males and females.

This is an excellent point and we completely agree that there would need to be an interaction of sex x session to justify the follow-up comparisons. We have now corrected this error. With new analyses, we did not find significant interactions thus we have eliminated any subsequent follow-up of “early vs. late sessions” and deleted former Figure 4 altogether. This makes for much simpler conclusions and interpretations in that there were sex differences that persisted in rewards collected and initiation omissions throughout all phases, in both modalities (new Figure 3).

2. It does not appear that males or females learned the stimulus-based contingencies in the probabilistic condition (and barely learned the initial task or the deterministic reversals). It’s not reported, but I would be surprised if performance is significantly different from chance in 2f, which makes comparison of learning less meaningful (i.e., if nobody learned then no one learned faster or slower). It’s stated in the methods that rats were given 10 sessions to reach criterion when learning the stimulus-based version of the task. The authors should show how many rats actually reached criterion and as mentioned above report whether performance differenced significantly from chance. I’m not sure that we can draw conclusions from these data at all unless there’s something interesting to deduce from the fact that female rats are more likely to omit/slower to initiate trials even in a condition where they have not learned the task.

The reviewer is absolutely correct that the rats do not learn the stimulus-based task well. This is similar to previous reports. We have added the following text to the description of results for stimulus discrimination: “Rats exhibited much more difficulty learning the stimulus-based task regardless of sex with only 65% of rats meeting criterion for initial discrimination learning within 10 sessions.”
And the following for stimulus reversal: “Unlike action-based reversals, animals exhibited difficulty learning stimulus-based reversals with only 44% reaching a 75% criterion on deterministic reversals, and only 15% of rats learning probabilistic reversals within 10 sessions.”
And the following in the Discussion: “All animals exhibited more difficulty learning the stimulus-based task indicated by accuracy maximally around chance level, similar to previous reports (9, 14). Only a handful of groups have conducted cross-modal comparisons of discrimination on reversal learning, finding that generally rats acquire olfactory associations faster than visual ones (19, 20). We think this learning domain asymmetry may have more to do with poor transfer of gains across sessions in visual-based learning, not a lack of trial-by-trial improvement within a session. Increasing the number of trials per session to 2x what we administered here is expected to greatly improve acquisition by amassing, not distributing, the learning.”
Although the majority of animals did not fully reach criterion on stimulus-based reversals, we still find it interesting that females omitted more trials and collected fewer rewards in both domains, even though they exhibited much better learning in the action-based task. This suggests that task engagement can be at least partially dissociated from learning.

3. I’m confused about the interpretation of the lapse rates in figure 5. It is stated in the text that e1 is larger than e2 which indicates that the rats make more errors prior to the transition. But doesn’t a higher lapse rate mean that they are selecting the worse offer less often (e.g., making fewer mistakes) than if they had a smaller lapse rate? Maybe the definition of errors in this case needs to be expanded a bit. I would think that every “worse” choice after the reversal is an error and the lapse rate prior to the behavioral transition captures the extent to which the animals are not making that choice.
We thank the reviewer for pointing this out. As explained in Eq. (7) in the Methods and illustrated in (re-labeled) Figure 4a, the sigmoid curve describes the probability of choosing the objectively better option, P(Better). So the reviewer is correct: the first lapse rate (before transition) indicates how much the animals “deviate” from choosing the worse option, which is P(Better) itself, whereas the second lapse rate (after transition) would measure how much they deviate from the better option (1−P(Better)). In other words, the ‘lapse’ rate in these transition curves specify the noise or the rate at which the current choice deviates from the assumed choice option for a given state. We think that the main confusion arises from the fact that the notion of an objectively “better” option is only available from the experimenter’s point of view and may not correspond to the animals’ subjective experience.

Therefore, to avoid any assumption about the accuracy of choice, we have avoided using the term ‘error’ and instead have edited the text as follows:
Overall, the lapse rates were significantly larger prior to the behavioral transition point compared to after the transition for both male (p = 3.56e-27) and female rats (p = 6.23e-24; mixed-effects regression on ε1 - ε2), consistent with the notion that rats’ choice behavior is more noisy before transitioning to the other choice option after reversal.

To better clarify the distinction between the notion of ‘lapse’ and ‘error,’ we have also added the following sentence in the Methods after Eq. (7) as follows: “Note that each lapse rate indicates the rate at which the animal’s choice deviates from the options assumed to be subjectively dominant during a given period, i.e., the worse option before transition and the better option after transition.”


4. I also don’t think that including the omit trials makes sense or if they are included, it doesn’t seem justified to interpret them as a choice halfway between right and left in this analysis. These are likely lapses of engagement and are therefore hard to interpret as a choice between the two options. It’s also a big assumption that the rats would have been equally likely to choose left or right if they had made a choice on those trials. There could be structure in the omissions (e.g., are they more likely after unrewarded trials? There is clearly structure in the choice across a session too in that they are more likely to make a the “better” choice late in the session). Including these trials in this way could also be a problem for interpretability given that females omit more trials than males. Are the differences between males and females really about how long females perseverate on the old option (which would lead to a larger post-transition lapse rate than males) or how often they omit? It’s also not clear how they were included anyway – the description of the model describes binomial data. Comparing the performance after the transition between males and females helps with the point the authors are trying to make (although the test is convoluted – why not just compare performance after the behavioral transition rather than comparing the differences in performance before and after the transition?)

We agree with the reviewer that our modeling choice for the omitted trials may have been based on strong assumptions. To address this concern, we have simply excluded the omitted trials from the fit to estimate the parameters. The statistical test of sex effect using a mixed-effects model still revealed a significant main effect of sex (βsex = 0.0524, p = .00496), such that females showed overall larger post-transition lapse rates compared to males.

As the reviewer has suggested, we also replaced the test to compare the performance of two groups after behavioral transition directly, finding significantly lower performance in females. Even more directly, we also found that the post-transition lapse rates (estimated after excluding the omitted trials from the fit) and the number of omissions after the estimated behavioral transition were significantly correlated, strongly in female rats (r = .527, p = 2.42e-10) and less strongly in male rats (r = .389, p = 5.79e-05). Therefore, we feel comfortable concluding that the female behavior is best characterized by both an increase in the omissions and post-transition lapse rates.

Accordingly, we have updated the relevant parts of the text as follows.

In the section ‘Differences in reversal learning identified through fitting of choice data’: “…analysis using mixed-effects GLMs revealed that there was a significant sex difference in the second lapse rate (ε2), with females exhibiting a higher lapse rate after the behavioral transition point compared to males (βsex = 0.0524, p = .00496; Fig. 4B). No significant main effect of sex was observed for the other parameters, after corrections for multiple comparisons (offset s: p = .0263; slope : p = .0803; first lapse rate ε1: p = .146). These results indicate that the behavioral tendency that most distinguishes males and females is the post-transition period. To confirm this possibility directly, we compared the performance of two groups after the behavioral transition point, estimated by the offset s (i.e., comparing P(correctTrials > s) within each session). We found a significant main effect of sex (βsex = -0.0293, p = .00972), with the female rats showing lower choice accuracy after transitioning to the better choice option. Repeating the same analysis for the number of initiation omissions (i.e., P(omitTrials>s)), we again found a significant sex difference (βsex = 0.091, p = 2.62e-06). Consistently, we found that the second lapse rates were significantly correlated with the number of omissions, strongly in females (r = .527, p = 2.42e-10), less so in males (r = .389, p = 5.79e-05).”

In Methods, after Eq. (8): “Omitted trials were excluded from the fit.”

For the omitted trials which were excluded from the fit, we instead sought to identify a potential structure in the omitted trials through additional analyses, per the reviewer’s suggestion. By running mixed-effects logistic regression on the trial-wise omission data, we indeed found a strong negative impact of previous reward feedback, such that rewards make omissions on next trial less likely. And while a main effect of sex was not significant itself (βsex = 0.250, p = .709), we found a significant interaction effect with trial number (βsex:trial = 0.00717, p = .00569), consistent with above results that female rats omit more trials toward later trials in a session.


5. The authors could do a better job justifying the behavioral modeling and why it is informative to compare them. Are they trying to argue that rather than incremental trial by trial updating (as captured with the RL model) the behavior is better explained as a state transition (as captured with the sigmoid fits)? I’m not sure that these hypotheses can be clearly distinguished in a deterministic task because once the animal starts choosing the high reward option they will always be rewarded so the value for that choice will never be reduced if it is chosen. So effectively, this would look like a transition. A test of this would be to use the RL model to simulate choices during reversals (so the mechanism of updating is known) and then compare performance of the two models on the simulated data. My guess is that they’d be pretty similar.
6. Line 459: poorer choice accuracy in females after the transition wasn’t tested

We thank the reviewer for this comment. Based on the quality of fit our conclusion was that, for these specific data, rat behavior is better described as a gradual transition in choices, rather than by a trial-by-trial update in response to reward feedback as the RL model assumes. Thus, the two models are most distinguished by their sensitivity to reward feedback. As the reviewer points out, the value of the better option in the RL model would not go down once the animal starts choosing that option. This would, in fact, predict a sharper transition and better performance than the sigmoid choice curve, because the first few trials would be sufficient for the RL model to discover the high-reward option.

To validate this claim and address the reviewer’s concern directly, we have simulated the choice data from two models (SC2 and RLdecay) during the deterministic task, using the estimated parameters from the data and repeating the simulation 100 times for each session. As expected, we found that the sigmoid transition curve is characterized by much more of a gradual transition compared to RL (new Fig.S4, below). This was reflected as significantly lower performance for the sigmoid functions compared to the RL model. Therefore, we find the RL model overestimates the animals’ learning and performance, even when optimized to the data.

It is of course possible that there could be models that better describe the rats’ behavior by utilizing the reward information, which we are not able to fully identify in the present study.

“From the comparison of simulated choice behavior, RL models overestimated the animals’ speed of transition and resulting performance (Fig. S4). This suggests that choice behavior is better described as a gradual transition, less sensitive to reward feedback than the RL model assumes. Focusing on the post-transition period, we found that females omitted more trials and exhibited poorer choice accuracy later in each reversal. Altogether, these findings suggest that females become more disengaged than males when there is not as much to learn.”


Supplementary Figure S4. Comparison of simulated choice behavior in the Action-based task using sigmoidal and RL models. (A) Plotted are averaged model-generated choice probabilities for the better option across simulated sessions (100/0 reward schedule), shown for the first 150 trials. The best respective model for sigmoid and RL were simulated (SC2 and RLdecay). Each deterministic session was simulated 100 times with the estimated parameters of each model. The RL model required only a few trials to reverse their choice option, overestimating the transition speed. (B) Comparison of simulated performance from two models. Sigmoid model had significantly lower performance (mixed-effects regression on paired difference in performance, (Psigmoid – PRL) ~ 1 + (1|SessionID); reported is the intercept β0 and its p-value). With faster transition to the better option, RL model overestimates performance.


Minor:
1. Is there a typo on line 195? Elsewhere it says the low probability option was rewarded 10% of the time rather than 0%. Thank you for flagging this, yes it was an error. For the probabilistic reversals, the low probability of reward was 0.10.

2. When describing the sigmoid fits at reversals, I think it would be clearer to refer to “behavioral transition” instead of just “transition” since this could be confused with reversal which has already happened. We thank the reviewer for this suggestion. We have now adjusted the wording to ‘behavioral transition’ in the text.

3. Fitting a sigmoid to choice around transitions isn’t a Hidden Markov Model. The paper that is cited used a Hidden Markov Model to infer transitions between states defined by the fit of the logistic regression around reversals (so the sigmoid fits were different in each state and the transitions between those states were described by the HMM rather than the transition from low to high choices at reversals). The reviewer is correct. We have now replaced the term with ‘sigmoidal transition curves.’

4. Was the sex*reversal interaction on omissions, trial initiation latencies and earned rewards described on line 369-371 (also, this is the sort of model design I described above – include the interaction and then use the specific post hoc comparisons to describe how the sex difference changes across conditions) assessed for the action-based task? As detailed above, we have now included session as a moderator in the GLM formulas and in no occasion was a significant interaction observed with sex. Additionally, the only instance of number of reversals influencing performance was in the latencies: as reversal experience increased, latencies were hastened in both males and females, yet there was no interaction of sex with reversal.

5. On line 399 the authors say that they do not include the stimulus-based sessions in the RL model fitting because the fit is poor but then there’s a section about it starting at line 436. Thank you for catching this. We have removed the statement in Line 399, because indeed there are some analyses presented in the later sections. We had meant to emphasize that, although fitting of choice behavior on stimulus-based task had been conducted, the quality of the fit was poor (especially given the animals’ poor performance), and therefore did not follow up with further analysis.


Reviewer: 2

Comments to the Author
The authors present new results comparing sex differences in decision making strategies. Here, the authors use a previously published paradigm to study sex differences during reversal learning in both an action and stimulus domain. Using mixed-effects models, reinforcement learning models, and state transition models, they find that female rats are more likely to omit trials, take longer to forget the value of unchosen options, and have a higher lapse rate after choice transitions, while male rats were more likely to utilize a Win-Stay strategy in action-based reversal learning.

Overall, I feel like the results of this study are a useful contribution to an under-explored area of animal decision making; however, I would suggest additional clarification and explanations to make the manuscript stronger.

High-level comments:

- The results section is sometimes lacking motivation for why specific measures are chosen. For example, why are initiation latencies, correct choice latencies, reward latencies, etc., being compared? What are these metrics meant to evaluate? Much of this could be bolstered by using a results format like: "First we wanted to test if X is different between male and female rats. To measure this, we used Y. The results show Z." This is already being done in some places, e.g. for "Action-based learning" on line 385, we see the motivation with the sentence: "To capture animals’ sensitivity to positive and negative feedback, we assessed their use of win-stay and lose-shift adaptive strategies in action-based reversal learning."

We thank the reviewer for this suggestion. We have now better justified the use of these specific measures and their analyses through the manuscript. For example, in the Introduction: “when fine-grained trial-by-trial data is collected, such behavioral methods allow us to gather information about attention, deliberation, and motivation through initiation, choice, and reward collection latencies, respectively, and to compute sensitivity to positive and negative feedback through Win-stay and Lose-shift metrics (9, 10)”

Similarly in the Results: “To assess the speed of initial acquisition, we used GLM formulas to determine the major predictors of this learning across both action and stimulus domains.”

Also in the Results: “We also assessed the influences of successful reward procurement across sex and in the two different domains.” And “We next assessed the influences on the initiation of trials, which is often used as a proxy for attention and task engagement.”

- There are some prior results mentioned in the introduction that seemingly conflict with some of the results seen in this study, e.g. female mice displaying more Win-Stay strategies in a two-arm bandit, and female mice being more sensitive to negative feedback. I don't think the disparities are problematic, but I believe it should be addressed and compared further in the results section.

Thank you for pointing this out. We do think this has to do with whether the task is stable or dynamic. We compare our findings of sex differences in win-stay and lose-shift in the discussion to previous studies, including the Chen paper.

- The conclusions feel fairly generic. It would be nice to have the conclusions be more tailored to what was discovered here, what questions it raises, how it fits into a broader picture, and how they would inspire follow up studies. It's unclear what chronic stress has to do with this paradigm, also there's the mention of previous studies' results showing sensitivity to negative feedback but no connection to what was seen in the present study.

We regret not having updated our discussion thoroughly prior to submission. We have deleted mention of chronic stress, and kept our interpretations and discussion of findings closer to the data.

- I leave this up to the authors' discretion, but I believe it would be useful to try applying a mixture-of-agents approach to modeling RL (e.g. Miller et al., 2017, Nat. Neuro.; Akam et al., 2021, Neuron) and comparing it with other potential strategies, such as a Win-Stay Lose-Shift or a perseveration strategy. Additionally, this might help provide a better model fit to the stimulus-based reversal learning task, where they don't seem to be using a RL strategy at all.

Thank you for this interesting suggestion. As addressed in our responses to Reviewer 1’s comments, we note that the rats do not learn the stimulus-based task well, and therefore the poor quality of fit for the stimulus-based task is expected (that is, for models that assume stimulus learning). Regarding heuristic strategies such as Win-Stay/Lose-Shift, we also note that stimulus-specific strategies are close to chance level as well (i.e., see Figure S2). Although it is possible that the animals during this task were relying on some other (non-stimuli specific) strategies or simply making random choices, we have refrained from exploring this possibility further as it is not the main focus of the present experiment. Therefore, instead of pinpointing potential strategies that the animals could have employed during this task through additional model fitting, we have emphasized the difficulty of learning the stimulus-based task compared to action-based one. To do so, we have added the following text to the description of results for stimulus discrimination: “Rats exhibited much more difficulty learning the stimulus-based task regardless of sex with only 65% of rats meeting criterion for initial discrimination learning within 10 sessions.”
And the following for stimulus reversal: “Unlike action-based reversals, animals exhibited difficulty learning stimulus-based reversals with only 44% reaching a 75% criterion on deterministic reversals, and only 15% of rats learning probabilistic reversals within 10 sessions.”
And the following in the Discussion: “All animals exhibited more difficulty learning the stimulus-based task indicated by accuracy maximally around chance level, similar to previous reports (9, 14). Only a handful of groups have conducted cross-modal comparisons of discrimination on reversal learning, finding that generally rats acquire olfactory associations faster than visual ones (19, 20). We think this learning domain asymmetry may have more to do with poor transfer of gains across sessions, not trial by trial improvements within a session. Increasing the number of trials per session to 2x what we administered here is expected to greatly improve acquisition by amassing, not distributing, the learning.”


Results-level comments:
- While I gleaned that the infusion was added to be able to include the additional control animals from a previous study, the results section lacks any mention of controlling for the role of infusions. Furthermore, the methods section states on line 106: "we entered drug as a covariate in order to account for any potential behavioral effects following CNO administration". However, the mixed-effects model later on does not include a factor for drug, so it is unclear how it is being controlled for in the main findings. Finally, it took a couple reads to really realize that it was only the control animals being used from the previous study, and I assume the injection protocol here is following the control protocol from the previous study, so I assume that the injections are not meant to have an effect. It would be nice for the purpose of the injections to be more clearly laid out, as well as to have a supplemental figure demonstrating that the infusion was controlled for and ideally does not impact the results seen in the main text.

We thank the reviewer for requesting this clarification, as it is an important one. Drug was included as a covariate in all GLM models, and we have now clarified its inclusion in the GLM model in the Data Analyses section to avoid confusion. In the methods section, we now more clearly note that all animals included in this experiment were control animals with a null virus in these brain regions, and that they were all injected intraperitoneally with either CNO or VEH counterbalanced across reversals. The effect of either injection is controlled for by including drug as a covariate in all our GLM models, which based on our analyses was never a significant predictor of learning or performance, and thus did not have an effect on any of our outcome measures.

- There are a number of trial-based metrics that would be strengthened by instead using or including trial count normalized metrics. For example, female rats were slower to initiate trials and collected fewer rewards, but is the latter because they are completing fewer trials? Is the reward rate per completed trial significantly different from males?

This is an excellent point and we did not clarify this enough. We now include the formulas in the Data Analyses section: Trials are included as a moderator in GLM models assessing learning for action-based reversals. We did not find a significant sex difference for either initial discrimination learning or reversal learning for the action-based task. However, we did find a sex difference in total number of trials completed in the stimulus-based task, and included trials in the analyses as a covariate for both initiation omissions and latencies to account for this.

- The learning curves for the stimulus-based task are somewhat concerning seeing as how they never surpass chance levels. Is this consistent with previous studies using this task? It would be useful to have a supplemental figure with the learning curve during training, since the criteria suggests that they need to reach at least 75% performance before a reversal. Although there is the additional statement in the methods that they were also advanced after 10 days -- did any of the rats every meet the 75% threshold?

The reviewer is absolutely correct that the rats do not learn the stimulus-based task well. This is similar to previous reports. We have added the following text to the description of results for stimulus discrimination: “Rats exhibited much more difficulty learning the stimulus-based task regardless of sex with only 65% of rats meeting criterion for initial discrimination learning within 10 sessions.”
And the following for stimulus reversal: “Unlike action-based reversals, animals exhibited difficulty learning stimulus-based reversals with only 44% reaching a 75% criterion on deterministic reversals, and only 15% of rats learning probabilistic reversals within 10 sessions.”
And the following in the Discussion: “All animals exhibited more difficulty learning the stimulus-based task indicated by accuracy maximally around chance level, similar to previous reports (9, 14). Only a handful of groups have conducted cross-modal comparisons of discrimination on reversal learning, finding that generally rats acquire olfactory associations faster than visual ones (19, 20). We think this learning domain asymmetry may have more to do with poor transfer of gains across sessions, not trial by trial improvements within a session. Increasing the number of trials per session to 2x what we administered here is expected to greatly improve acquisition by amassing, not distributing, the learning.”
Although the majority of animals did not fully reach criterion on stimulus-based reversals, we still find it interesting that females omitted more trials and collected fewer rewards in both modalities, even though they exhibited much better learning in the action-based task. This suggests that task engagement can be at least partially dissociated from learning.


Line-specific comments:
We thank the reviewer for their kind attention to detail. See below.
- Line 153: use either "minutes" or add a period for the abbreviation "min." Added
- Line 228: missing closing bracket. Added
- Line 365: typo "continency". Corrected
- Line 399: Statement about stimulus-based reversal learning is redundant with following section. Corrected
- Line 436: underscore after paragraph heading. Removed

Round 2 reviews
Reviewer 1
Recommendation – Minor Revision
Comments to author:
The authors addressed my comments and the clarified and improved statistical analysis has improved the manuscript. There are still some places where the descriptions are unclear or conclusions are not supported by the specific test. Overall, my comments are mostly minor. The weakest part of the manuscript remains the section about the RL modeling and sigmoid fits. Details are below


1. I think that the manuscript would be improved by slightly different framing of the section starting on line 403. I don’t think competing the models against each other is actually very useful in this case and it might be more effective to emphasize what the different approaches can reveal rather than trying to claim one is better than the other. The RL model could be used to identify potential differences in the use of reward feedback, and I like the idea of using the sigmoid fits to estimate the behavioral transition after the reversal. The differences in lapse rates between males and females following this transition is interesting. But the idea that a model comparison with RL tells us anything about mechanism is weak. The sigmoid fits don’t make any predictions about mechanism. How distinguishable is an RL mechanism and a “gradual shift in preferred options” (line 514) as the sigmoid fit is described? A couple of other related points
a. Line 515 still references a Hidden Markov Model as inspiration for this analysis. While I understand that the authors took inspiration for this analysis from the model in the referenced paper, the sigmoid fits were not used to define state transitions in that paper, they were used to define the states themselves, and the transitions were modeled with the HMM.
b. The title for the section on 403 “Differences in reversal learning identified through fitting of choice data” could be more specific and informative. Many of the preceding statistical tests involve “fitting of choice data”. It would be nice to use the title to tell us more specifically what the model fitting here is accomplishing or broadly what the differences were
2. Line 440: You can’t say that an effect is stronger in one group than the other because of different p-values. This would need to be explicitly compared. It’s not clear why this matters though. It would be helpful for the authors to provide some interpretation of this result. What is the significance of a correlation between lapse rate and omitted trials?
3. Line 234 says that session was included as a covariate, but in the formula, session is not named.
4. Line 322 states that BIC was used to assess model fit, but everywhere else AIC is described
5. It is unclear why the authors decided to display the data differently for the action-based and stimulus-based learning data in Figure 2. If it is easier to see the change in performance between session 1 and 5 when averaged across trials, it makes the most sense for the authors to show the data both ways for both experiments. Omitting the trial-based plotting of the stimulus-based data also makes it impossible to assess this statement in the discussion, the basis of which is unclear (Line 470): “We think this learning domain asymmetry may have more to do with poor transfer of gains across sessions in visual-based learning, not a lack of trial-by-trial improvement within a session.” (note also that visual-based learning rather than stimulus-based learning is here)

Reviewer 2
Recommendation – Accept
Comments to author:
I believe the authors have adequately responded to the previous round of reviews, and I believe their edits have strengthened the clarity of the manuscript. I have no additional major comments, but have a few minor formatting suggestions:
- line 81: “based on a sigmoidal transition curves” - either remove the “a” or remove the “s” off curved
- the equation number is missing for the first equation (line 261), and the other numbers are a bit close to the other equations, making them appear as if a part of the equation
- line 280: there is a stray “/“ at the end of the equation
- line 296: there is a stray “+” at the end of the equation
- there is inconsistent use of a leading 0 in the fractional metrics. E.g. line 337 uses a leading 0 (0.205) but line 432 does not (.0803 and .146)

Decision letter
Thank you for submitting your revised manuscript OXFNSC-2025-003.R1 - "Sex differences in task engagement and lapse rate during reward learning" - to Oxford Open Neuroscience.

Your manuscript was re-reviewed by the original two reviewers. I’m grateful for their detailed and helpful comments.

Both reviewers agree that you have responded to the issues raised in the previous round of reviews. I am issuing a ‘minor revision’ decision to give you the opportunity to respond to their comments below, however I will not make this a formal condition for acceptance. Once I receive a revision I will endeavour to deal with it swiftly, without further review.

I invite you to revise your manuscript within the next 30 days from today’s date.

Please note that this journal operates with transparent peer review. This means that if your submission is accepted for publication, the full peer review history of your article will publish online alongside your article. This includes reviewer comments, editor decision letters, and your author responses.

To revise your manuscript, log into https://mc.manuscriptcentral.com/oxfnsc and enter your Author Centre, where you will find your manuscript title listed under "Manuscripts with Decisions."  Under "Actions," click on "Create a Revision."  Your manuscript number has been appended to denote a revision.

You may also click the below link to start the revision process (or continue the process if you have already started your revision) for your manuscript. If you use the below link you will not be required to login to ScholarOne Manuscripts.

*** PLEASE NOTE: This is a two-step process. After clicking on the link, you will be directed to a webpage to confirm. ***

https://mc.manuscriptcentral.com/oxfnsc?URL_MASK=359b612a128f44199f6e23c46f2029af

You will be unable to make your revisions on the originally submitted version of the manuscript.  Instead, revise your manuscript using a word processing program and save it on your computer.  Please also highlight the changes to your manuscript within the document by using the track changes mode in MS Word or by using bold or colored text.

REMINDER: if you have not already done so, when submitting your revised manuscript, please include a short alternative-text description under the figure legend for each figure. Alt-text is required to ensure the accessibility of our publications. Guidance is available on the journal’s Author Guidelines webpage and at https://academic.oup.com/pages/for-authors/journals/preparing-and-submitting-your-manuscript

Once the revised manuscript is prepared, you can upload it and submit it through your Author Centre.

When submitting your revised manuscript, you will be able to respond to the comments made by the reviewer(s) in the space provided.  You can use this space to document any changes you make to the original manuscript.  In order to expedite the processing of the revised manuscript, please be as specific as possible in your response to the reviewer(s).

IMPORTANT:  Your original files are available to you when you upload your revised manuscript.  Please delete any redundant files before completing the submission.

Because we are trying to facilitate timely publication of manuscripts submitted to the Oxford Open Neuroscience, your revised manuscript should be uploaded within the next 30 days from todays date.  If it is not possible for you to submit your revision in a reasonable amount of time, we may have to consider your paper as a new submission.

Once again, thank you for submitting your manuscript to the Oxford Open Neuroscience and I look forward to receiving your revision.

Authors’ response
Reviewer: 1

Comments to the Author
The authors addressed my comments and the clarified and improved statistical analysis has improved the manuscript. There are still some places where the descriptions are unclear or conclusions are not supported by the specific test. Overall, my comments are mostly minor. The weakest part of the manuscript remains the section about the RL modeling and sigmoid fits. Details are below

1. I think that the manuscript would be improved by slightly different framing of the section starting on line 403. I don’t think competing the models against each other is actually very useful in this case and it might be more effective to emphasize what the different approaches can reveal rather than trying to claim one is better than the other. The RL model could be used to identify potential differences in the use of reward feedback, and I like the idea of using the sigmoid fits to estimate the behavioral transition after the reversal. The differences in lapse rates between males and females following this transition is interesting. But the idea that a model comparison with RL tells us anything about mechanism is weak. The sigmoid fits don’t make any predictions about mechanism. How distinguishable is an RL mechanism and a “gradual shift in preferred options” (line 514) as the sigmoid fit is described?

We thank the reviewer for this thoughtful point. Regarding the distinction between the two types of models, we had presented a supplementary analysis in Figure S4 showing that the two models make statistically distinct predictions about behavioral transitions, and judging from quality of fit (AIC), rats’ behavior is better described by the sigmoid function than RL models. However, we do agree with the reviewer that the sigmoid function lacks a concrete mechanism, and therefore it may not be useful to directly compare with RL models. To clarify this point, we have taken care not to make any claims about the superiority of any model in explaining the behavior. Instead, we simply provide objective comparisons of the AIC metric and that the sigmoidal function simply “better described” the rats’ transition behavior (Line 417). In the Discussion section, we have also added a statement that these two models capture different aspects of behavior and need not compete with each other.
Line 524: “However, these results alone do not necessarily rule out the presence of error-driven learning like RL, as sigmoid transition curves lack concrete mechanisms for generating choice behavior. Rather, these two types of models capture different aspects of behavior. Future modeling studies could draw on both types of models to examine more nuanced mechanisms that consider the effect of task disengagement on learning and choice. This could directly address, for example, whether choice omissions and lapse trials are generated from the same internal state.”
A couple of other related points
a. Line 515 still references a Hidden Markov Model as inspiration for this analysis. While I understand that the authors took inspiration for this analysis from the model in the referenced paper, the sigmoid fits were not used to define state transitions in that paper, they were used to define the states themselves, and the transitions were modeled with the HMM.
We have now replaced the term as “the previous study” to avoid any confusion.
b. The title for the section on 403 “Differences in reversal learning identified through fitting of choice data” could be more specific and informative. Many of the preceding statistical tests involve “fitting of choice data”. It would be nice to use the title to tell us more specifically what the model fitting here is accomplishing or broadly what the differences were.
We thank the reviewer for this suggestion. While it is true that the preceding mixed-effects GLMs also involve optimization to choice data for estimating the parameters, their response variables were mainly summary statistics or probability measures computed over multiple trials data (e.g., accuracy, choice omissions, win-stay/lose-switch). Therefore, what we had meant to emphasize by “fitting of choice data” was mainly the fact that RL and sigmoid models aim to capture actual choice data on a trial-by-trial basis. To make this point clear and highlight the main findings, we have adjusted the section tile as Fitting of trial-by-trial choice data reveals sex differences in lapse rates.
2. Line 440: You can’t say that an effect is stronger in one group than the other because of different p-values. This would need to be explicitly compared. It’s not clear why this matters though. It would be helpful for the authors to provide some interpretation of this result. What is the significance of a correlation between lapse rate and omitted trials?
We thank the reviewer for pointing this out. We had previously relied on the larger correlation coefficient, rather than its p-value, to claim that the correlation is stronger in females (r=0.53) than in males (r=0.39). Yet, we acknowledge that a direct statistical test has not been done to conclude any group difference. Using the Fisher’s r-to-z test, we find that the difference in the correlation coefficient is not statistically significant (p=0.328) given our current sample size. We therefore now simply state that the correlations are significant in both female and male rats. We have now added this interpretation in Line 441: “This result is consistent with the interpretation that post-transition disengagement is a common state that underlies both lapse and omitted trials.”
3. Line 234 says that session was included as a covariate, but in the formula, session is not named.
We thank the reviewer for pointing this out; the reviewer is correct that session was not included as a covariate in the model for performance measures (i.e. omission, latencies, rewards collected). Our original analysis involved splitting some of the performance measures (i.e. initiation omissions, rewards collected) by session, which is why it was originally included as a covariate. However, it was pointed out in the previous round of revisions that session should not be analyzed separately unless there was an interaction with sex. Given that there was no significant interaction with sex, we re-ran these analyses using outcome measures that were either averaged or summed across sessions. Thus, session was not included in the GLM formulas for any of the performance measures in the final set of analyses. We have made that clear in the text now (Line 234).
4. Line 322 states that BIC was used to assess model fit, but everywhere else AIC is described We thank the reviewer for this attention to detail. The note about BIC in Line 322 was specific to mixed-effects GLM models, as a reference to our previous statement in Line 222-223: “For selection of the best-fitting model, we used the GLM formula with the lowest Bayesian Information Criterion (BIC).” However, for RL and sigmoid-based models that aims to capture trial-by-trial choice data, we mainly employed AIC.
We have now clarified this distinction in Line 316 and Line 321.
5. It is unclear why the authors decided to display the data differently for the action-based and stimulus-based learning data in Figure 2. If it is easier to see the change in performance between session 1 and 5 when averaged across trials, it makes the most sense for the authors to show the data both ways for both experiments. Omitting the trial-based plotting of the stimulus-based data also makes it impossible to assess this statement in the discussion, the basis of which is unclear (Line 470): “We think this learning domain asymmetry may have more to do with poor transfer of gains across sessions in visual-based learning, not a lack of trial-by-trial improvement within a session.” (note also that visual-based learning rather than stimulus-based learning is here).
We thank the reviewer for this suggestion. We have now modified the figure so that accuracy for both action- and stimulus-based learning is shown across trials for the first and last session for both domains. Additionally, the GLM model for stimulus-based learning accuracy was re-run to include trials as a moderator. This was done for consistency to match the analyses that were conducted for action-based learning, and also to more accurately reflect the updated figure and the statement above. Both action- and stimulus-based learning showed improvements across session and trials, though much slower for stimulus-based learning.


Reviewer: 2

Comments to the Author
I believe the authors have adequately responded to the previous round of reviews, and I believe their edits have strengthened the clarity of the manuscript. I have no additional major comments, but have a few minor formatting suggestions:
We thank the reviewer for their attention to detail. Please see below.
- line 81: “based on a sigmoidal transition curves” - either remove the “a” or remove the “s” off curved. Removed “a” from the sentence.
- the equation number is missing for the first equation (line 261), and the other numbers are a bit close to the other equations, making them appear as if a part of the equation.
We were not sure if this is a file conversion issue, as we do see equation number (1) and the formatting is consistent across all equations. We have double-checked that all equations appear correctly.
- line 280: there is a stray “/“ at the end of the equation.
We were not sure if this is also a file conversion issue, but there should have been ‘N’ after the division sign (“/”), which refers to the number of trials in each session. We have double-checked that the equation appears correctly.
- line 296: there is a stray “+” at the end of the equation.
We were not sure if this is a file conversion issue, but there should have been ‘ϵ’ term after “+” sign. We have double-checked that the equation appears correctly.
- there is inconsistent use of a leading 0 in the fractional metrics. E.g. line 337 uses a leading 0 (0.205) but line 432 does not (.0803 and .146).
We have modified fractional metrics to be more consistent throughout the manuscript.

Associate Editor

Comments to the Author:
(There are no comments.)
Round 3 
Final Decision Letter
It is a pleasure to accept your revised manuscript entitled "Sex differences in task engagement and lapse rate during reward learning" in its current form for publication in the Oxford Open Neuroscience.  

Please note that this journal operates with transparent peer review. This means that  the full peer review history of your article will publish online alongside your article. This includes reviewer comments, editor decision letters, and your author responses.

Next steps
You will receive an email from no-reply@scipris.com within roughly one week. This is your invitation to sign up for an account with SciPris, Oxford University Press’ author portal hosted by Aptara. You will need to create an account if you do not already hold one. Please register or log into your account and follow the online instructions which will guide you through signing your licence and paying the APC. The email and the portal have clearly signposted support options if you need any help during this process.

Please note that SciPris is a completely different system from ScholarOne, so your credentials to submit your manuscript here will not work there. Once you’ve created a SciPris account, you will be able to use it whenever you publish with Oxford Open Neuroscience or any OUP journal. Please note that OUP will only ever request payment for applicable fees be made via SciPris or to an OUP bank account. If you ever have concerns about the legitimacy of a request, please do not hesitate to contact a customer services agent via the SciPris portal or directly via oupsupport@scipris.com.

Thank you for your fine contribution.  On behalf of the Editors of the Oxford Open Neuroscience, we look forward to your continued contributions to the Journal.
